# Supplementary material for: Association between oral microbiome and seven types of cancers in East Asian population: a two-sample Mendelian randomization analysis
Source: Front Mol Biosci. 2023 Nov 21;10:1327893. doi: 10.3389/fmolb.2023.1327893 (PMC10702768; doi:10.3389/fmolb.2023.1327893)
Supplement: Supplementary file 1 [file DataSheet1.ZIP › Supplementary Materials/MR plots for tongue/tongue═╝/Pancreatic cancer/╥╚╧┘░⌐_╔╕╤í╜ß╣√/pheno.228_to_pancreatic cancer_leave_one_out.pdf]

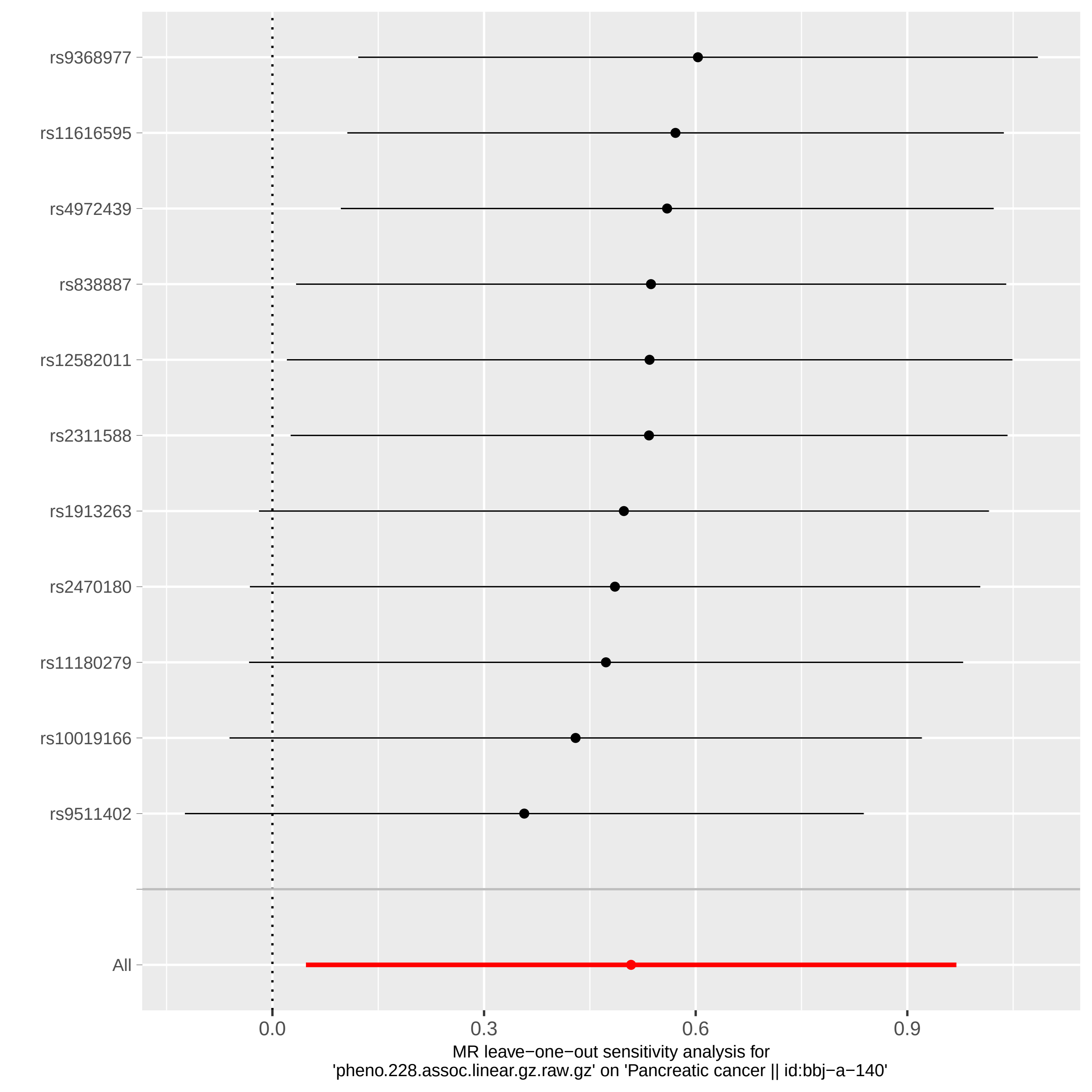
rs9368977
rs11616595
rs4972439
rs838887
rs12582011
rs2311588
rs1913263
rs2470180
rs11180279
rs10019166
rs9511402
All
0.0
0.3
0.6
0.9
MR leave−one−out sensitivity analysis for
'pheno.228.assoc.linear.gz.raw.gz' on 'Pancreatic cancer || id:bbj−a−140'
